# Reanimation Techniques of Peripheral Facial Paralysis: A Comprehensive Review Focusing on Surgical and Bioengineering Approaches

**DOI:** 10.3390/jcm13206124

**Published:** 2024-10-14

**Authors:** Carmelo Saraniti, Barbara Verro

**Affiliations:** Division of Otorhinolaryngology, Department of Biomedicine, Neuroscience and Advanced Diagnostic, University of Palermo, 90127 Palermo, Italy; carmelo.saraniti@unipa.it

**Keywords:** facial paralysis, facial injuries, facial reanimation, reconstructive surgery

## Abstract

Peripheral facial paralysis represents a disabling condition with serious psychological and social impact. Patients with peripheral facial paralysis have a disfigurement of the face with loss of harmony and symmetry and difficulties in everyday facial functions such as speaking, drinking, laughing, and closing their eyes, with impairment of their quality of life. This paralysis leads to impairment of facial expression, which represents one of the first means of communication, an important aspect of human interaction. This review aims to explore the reanimation techniques for managing peripheral facial paralysis. An analysis of static and dynamic techniques for facial reanimation is provided, including muscle flaps, nerve grafting techniques, and bioengineering solutions. Each technique showed its benefits and drawbacks; despite several options for facial reanimation, no technique has been detected as the gold standard. Therefore, each patient must be evaluated on an individual basis, considering their medical history, age, expectations, and treatment goals, to find the best and most fitting treatment.

## 1. Introduction

Peripheral facial paralysis (PFP) consists of the paralysis of one side of the face. Bell’s palsy represents the main cause of PFP [1]. Other causes may be infection, trauma, tumor, and surgery [2].

It leads to a change in facial expression, which represents an important form of non-verbal communication. As stated by Rubin, facial expressions are the mirror of our inner emotions [3]. This concept could be summed up in the words of the founder of hand surgery, Sterling Bunnell (1927): “The delicate shades of continence are lost. Joy, happiness, sorrow, shock, surprise, all the emotions have for their common expression the same blank stare”.

Moreover, peripheral facial paralysis causes difficulties in swallowing, drinking, and speech and may cause eye problems due to failure to close the eyelid. All these impairments result in a worsening of the quality of life [4]. Therefore, the main goals of facial reanimation surgery are to recover, as much as possible, facial symmetry and tone. Over the years, different strategies and therapies have been attempted and developed to deal with this disability and to revive the facial nerve. The first attempt was performed by Sir Balance in 1895. Then, other surgeons tried to develop new surgical strategies. Among the surgical techniques, some aim to recover the motility of the lower third of the face, others the motility of the upper third of the face by using local or free muscle flaps. In some cases, where conditions permit, the attempt was made directly on the facial nerve.

The techniques of facial nerve reanimation can be distinguished into static and dynamic. As well explained by McLaughlin (1949), both supports need a static one at rest and dynamic support during facial movement. Obviously, static techniques are easier to perform but cannot ensure the best facial recovery on their own. McLaughlin also suggested preferring static support for the forehead and eyelid and dynamic support for the lower third of the face [5]. About dynamic techniques, whenever possible, nerve surgery provides the best results in comparison with muscle transplantation. As stated by Boahene [6], the more time that passes after denervation, the lower the chance of reinnervation and recovery.

This review attempts to summarize what has been studied and researched so far for facial reanimation, with a glance to the future.

By analyzing the current and most used grading scales for PFP, the review separately explores the techniques of resuscitation of the lower third and upper third of the face. The fourth part of the review deals with nerve reconstruction techniques, and in the last part, the future perspectives are explored, focusing on bioengineering approaches.

## 2. Facial Nerve Grading System

Many facial nerve classifications could be used: the House–Brackmann Grading System (HBGS) [7], the Sunnybrook Facial Grading System (SFGS) [8], the Facial Clinimetric Evaluation (FaCE) scale [9], and the Facial Disability Index (FDI) [10].

HBGS was introduced in 1985 by the Facial Nerve Disorders Committee of the American Academy of Otolaryngology-Head and Neck Surgery. It is performed by the physician who assesses the facial appearance at rest, its residual movements, and synkinesis, if present. Like HBGS, the SFGS, developed by Ross et al. in 1992 [11], evaluates the same parameters in terms of facial symmetry at rest and during movement and synkinesis. The FaCE scale consists of 50 items administered to patients to measure the patient’s perception of facial disability and impairment. Kahn et al. demonstrate that the FaCe scale should be used along with physician-grade scales (e.g., HBSG or FGS) to achieve a complete analysis of PFP [9]. FDI is a self-administered test consisting of 10 items that evaluate facial disability and its impact on social life.

However, to date, there is not a gold standard classification. Indeed, the degree of movement impairment or asymmetry could be objectively evaluated. On the contrary, the patient’s facial disability is very variable from patient to patient based on different factors, first, due to different levels of facial expression. In this regard, in 1974, Rubin [3] stated that the existence of several facial muscles leads to different expressions and defined three main types of smiles: the “Mona Lisa” smile exposing the upper teeth, the “canine” smile showing the canine teeth, and the “full denture” smile showing both upper and lower teeth.

## 3. Reanimation of Lower Third of Face

Surgery for reanimation of the lower face aims to enable spontaneous smiling as well as volunteer mouth movements, correct swallowing, and talking.

### 3.1. Fascia Lata Graft

As described by McLaughlin [5], the fascia lata graft can be used as a static technique for lower third face reanimation. He considered fascia lata, the iliotibial tract, as the best since it is flattened and used for tension. A vertical incision is performed on the lateral side of the thigh, the subcutaneous tissue is removed, and the proper length of the fascia lata is excised. Once performed, facial skin and the superficial musculoaponeurotic system (SMAS) dissection, the procedure consists of fastening the fascia lata at two points: the corner of the mouth (upper and lower lips, modiolus) and temporal fascia or malar bone. The author suggested the overcorrection since a stretch will occur post operatively. This technique showed satisfactory results, ensuring symmetry of the lower third of the face and oral competence in all patients. The authors reported no need for graft-related revision surgery and only one case of preuricular wound infection and dehiscence [12,13].

### 3.2. Lengthening Temporalis Myoplasty

This technique consists of the use of the temporalis muscle as a graft for facial reanimation, both for buccal and orbital paralysis. The first surgeons who proposed this technique were Gillies (1934), who used a portion of the temporalis muscle [14], and McLaughlin (1946), who suggested using the entire muscle [5]; in both cases, a fascia lata flap was also needed.

To restore smile, as reported by Labbè et al., the temporalis muscle tendon is transferred from its coronoid process attachment to the labial commissure and sutured to the upper lip levator muscles (e.g., levator labii superioris muscle, alae nasi muscle) [15,16,17]. As explained by Al Khabori et al. [18], attaching the tendon fibers (that correspond to the posterior part of temporalis muscle) to the commissure ensures a horizontal traction vector.

Post-operative complications may be hematoma, infection both on the surgical site and in the temporalis muscle tendon, and tendon dehiscence [18]. Rehabilitation usually lasts one year; in the end, the patient has a symmetric smile without effort and a spontaneous smile in some cases.

### 3.3. Latissimus Dorsi Muscle Flap

The technique consists of harvesting and splitting the latissimus dorsi muscle flap, including the transverse and descendent branches of the thoracodorsal vessels and the thoracodorsal nerve. So, a skin incision is performed about 2 cm beyond the nasolabial fold of the paralyzed side; once the pocket is created, the muscle flaps are placed and anchored to the periosteum of the zygomatic bone. Nerve anastomoses are performed differently between the two muscle flaps: the flap placed higher (allowing spontaneous smiling) will be innervated thanks to anastomosis between the branch of the thoracodorsal nerve and the buccal branch of the contralateral facial nerve, while the flap placed closer to the corner of the mouth (allowing voluntary smiling) will be innervated by the ipsilateral masseteric motor nerve. Flaps vascularization is achieved thanks to anastomosis between thoracodorsal vessels and facial vessels. This procedure has been shown to achieve both spontaneous and voluntary smiling. The authors developed this technique based on previous similar surgeries: neurovascular latissimus dorsi flap innervated by the contralateral facial nerve [19], gracilis muscle flap innervated by the ipsilateral masseteric nerve [20] with or without a second innervation by contralateral facial nerve [21]. The first technique allowed only spontaneous smiling, while the second one allowed only voluntary smiling. The third technique, reported by Biglioli et al. [21], resulted in the recovery of both spontaneous and voluntary smiling, as well as the technique by Okazaki et al. [22]. However, these last authors stated that their procedure has two main advantages: first, it does not need nerve grafting (Biglioli used sural nerve graft), and the results are more predictable. About this surgery, Takushima et al. suggest using the ipsilateral trigeminal nerve for the neuroanastomosis instead of the contralateral facial nerve in order to reduce the risk of weak cheeks if they are fatty [23].

### 3.4. Sternohyoid Muscle Flap

First described by Alam et al. [24], the sternohyoid muscle flap represents a valid alternative to the gracilis muscle flap in facial nerve reanimation. Indeed, its dimensions are like zygomatic muscle size in comparison with the gracilis, which is bigger with consequent facial dysmorphism. Moreover, it has a single pedicle consisting of a lingual or superior thyroid artery, a branch of the jugular vein, and the most powerful branch of the ansa cervicalis. This flap is sutured to the corner of the mouth and to the zygomatic periosteum and nerval anastomosis is performed with the masseteric nerve. Despite being a small scientific study, Suné et al. [25] demonstrated that this technique enables quite natural facial movement recovery and minimum morbidity to the donor site.

## 4. Reanimation of Upper Third of Face

Eyelid surgery should have these main goals: narrowing the palpebral fissure to reduce the lagophthalmos and the risk of epiphora, as well as conjunctivitis and corneal ulceration [26].

### 4.1. Masseter Muscle Flap

The masseter muscle is the main chewing muscle. It can be used for muscle transposition, both for the angle of the mouth and for the canthus of the eye [27,28]. In this latter case, the procedure consists of splitting the muscle belly, leading to possible nerve injury: only one-third of the masseter muscle needs for transposition. To prevent this complication, Shinohara et al. [29] proposed to elongate the muscle by using the fascia lata. The authors demonstrated the efficiency of this procedure with good outcomes and patient satisfaction.

### 4.2. Lateral Tarsal Strip (LTS)

First described by Anderson et al. [30], the technique consists of a lateral canthotomy and cantholysis and the splitting of the eyelid into posterior and anterior lamellae. After removing a marginal triangular area from the anterior lamella, lateral tarsal strips (upper and lower) are created, sutured together, and anchored to the periosteum, close to the Whitnall tubercle.

This surgery provides many advantages, such as the absence of sutures in the marginal lid, no risk of phimosis, reduced risk of laxity of canthal tendon over time, quick surgery, and the preservation of natural canthal angle.

However, in case of laxity of the upper eyelid, this procedure may cause the overlapping of the upper eyelid on the lower one. In this case, the combination with lateral tarsorrhaphy is suggested [31].

### 4.3. Lateral Tarsorrhaphy

This static procedure is performed to correct the ectropion. It consists of elevating the lower eyelid. However, as stated by Kwon et al., this procedure alone does not ensure good cosmetic results, so the authors suggest also performing a lateral tarsal strip [32]. Therefore, after performing the LTS, a portion of the mucocutaneous junction in the upper eyelid is removed, and a needle is passed from the tarsal strip to the lateral orbital rim (internal side), just above the canthal tendon insertion and, in the end, sutured to the lateral upper lid margin. This combined technique ensures the correction of ectropion as well as satisfying cosmetic results [33]. However, it may lead to complications such as infection, suture dehiscence, and a few cases of phimosis [31].

### 4.4. Tessier’s Technique

Tessier’s technique (1969) should be performed for the correction of lagophthalmos [34]. The procedure consists of the elongation of the levator muscle of the upper eyelid by the interposition of the temporal aponeurotic graft to avoid the upper eyelid levator muscle weakening [35]. Several studies demonstrated the efficacy of this surgical procedure in achieving complete or near palpebral closure, in addition to ensuring eyesight and good aesthetic results. No occurrences of keratitis or conjunctivitis were reported during the observation period [36,37,38].

### 4.5. Conchal Cartilage Graft

In this case, under general anesthesia, a graft of conchal cartilage (preferably from the same side as the paralysis) is harvested and placed into the lower eyelid. The perichondrium on one side of the conchal cartilage must be preserved in order to ensure its vascular support. After shaping the graft, it is placed into a pocket created under the tarsus without sutures. The use of autograft represents an advantage of this procedure, as well as the easy harvesting of the graft, good cosmetic results on the donor site, and the pliability of conchal cartilage compared to septal or costal cartilages. The authors reported few post-operative complications, such as hematoma and slight entropion. The main drawbacks are the possible visibility of the graft in the case of thin skin and the need to bend the neck to look down [39].

### 4.6. Autologous Dermis Graft

A rectangular dermal graft is harvested from the supraclavicular area or iliac crest. The height of the graft must be twice that of the retraction of the lower eyelid. The graft is placed and sutured in a pocket between the tarsus and the lower lid retractors.

A recent study compared the two types of autologous grafts: conchal cartilage and dermis [40]. The authors demonstrated that they are both efficient. However, the former is related to the risk of donor site complications, e.g., hematoma and keloid. On the contrary, the dermal graft is stiffer and more available. Moreover, it could be harvested together with the fat to compensate for the lower lid lipoatrophy.

## 5. Nerve Reconstruction

### 5.1. Trifurcation Approach

As described by Beutner et al. [39], the so-called “trifurcation approach” consists of the use of two nerves: the greater auricular nerve (GAN) and the hypoglossal nerve. This technique is based on previous studies that demonstrated the benefits of GAN as a graft in facial nerve reconstruction, sometimes used to cover a gap [41,42]. However, its enforcement is limited to only one neural branch defect [43]. In these cases, following the “combined approach” by Stennert [44], Beutner et al. suggest using the GAN graft to reanimate the upper face: the trunk of the GAN graft is sutured to the mastoid segment and its branches to temporal and zygomatic branches of the facial nerve by end-to-end suture. The hypoglossal nerve is sutured to the marginal branch of the facial nerve by endo-to-side suture, while the descending branch of the hypoglossal nerve is sutured end-to-end to the facial buccal branch. The use of the hypoglossal nerve has proven to allow better muscle tone and voluntary movement of the lower midface (e.g., spontaneous smile), without triggering involuntary tongue movements. First described by Korte et al. in 1903 [45], the original end-to-end hypoglossal-facial nerve transfer (HFNT) technique has been revised a few times over the years with the suggestion of the partial end-to-end HFNT with or without the use of a bridge graft, the “jump” procedure [46], or the modified partial procedure [47]. Le Clerc et al. compared these three techniques, finding good results in all cases, with the best outcomes in the modified partial procedure [48]. The same results were also found by Venail et al. [49], with only a few cases of tongue hemiparesis. In 2011, Volk et al. [50] attempted facial reconstruction by using the “jump” procedure along with facial nerve interpositional graft. Doing so, they demonstrated a lower incidence of hemitongue hypotrophy as well as lower cases of synkinesis between the lower and the upper face muscles, stressing the importance of the graft. However, regardless of the technique performed, to date, the HFNT represents the most used technique.

### 5.2. Masseteric Nerve Transfer

To avoid the consequences on lingual motility due to HFNT, masseteric-facial nerve anastomosis was proposed [47,51]. The masseteric nerve is a branch of the trigeminal nerve, and it is easily accessible during the procedure for facial nerve exposure. Once the nerves are accessed, the GAN graft is used to cover the gap between the masseteric and facial nerves. Indeed, in the case of complete PFP, the masseteric nerve is sutured to the facial nerve trunk by interpositioning of the graft [52,53]. In the case of partial facial paralysis, the masseteric nerve could be anastomosed to the zygomatic branch of the facial nerve [54]. However, this procedure does not enable the spontaneous smile, but patients achieve facial symmetry and voluntary smile, with minimum donor site aesthetic impairment [55].

### 5.3. Cross-Face Nerve Grafting

It is a two-step technique that includes the use of the contralateral facial nerve as a donor nerve graft and the harvesting of free flaps (e.g., pectoralis minor, gracilis, latissimus dorsi) in the paralyzed side of the face [56,57]. This technique, first described by Scaramella et al. (1971) [58] with the use of a sural nerve graft, was then modified by Harii et al. (1976) [59] by covering the sural nerve graft with the gracilis muscle. The surgery consists of harvesting the sural nerve graft that is sutured with the zygomatic branch of the contralateral healthy facial nerve and positioned into a pocket created in the gingivobuccal sulcus where the graft is anastomosed with the transected facial nerve. According to the technique described by Peng et al. [60], during the second-stage surgery (after about 6 months), the free edge of the sural nerve (in the gingivobuccal sulcus) is sutured with the obturator nerve of the gracilis free muscle. This procedure has many advantages such as good muscular contraction as well as coordination of oral movements (e.g., smiling) thanks to the control of the same functioning facial nerve. Moreover, it is related to minor morbidity of the donor site and to a mild risk of lesion of the healthy facial nerve during dissection. However, it requires a long learning curve to achieve good results [57,60].

## 6. Future Directions

In the last decades, bioengineering approaches have been proposed for facial nerve reanimation, and preclinical studies on animal models have been carried out [61]. In particular, some authors described the use of a silicone conduit embedded with fibroblast growth factor (FGF-2) [62] or adipose stem cells [63] in a rat facial palsy, demonstrating the potential regenerative role of the facial nerve. Also, biodegradable conduits (e.g., type I collagen, poly(glycolic acid)) embedded with bone marrow stem cells or adipocytes enable nerve regeneration, with similar results of autologous nerve grafting [64,65]. Three-dimensional (3D) technologies could also be used to customize nerve guidance conduits (NGCs) [66,67] that integrate with growth factors and/or cells, which may help nerve repair and recovery.

In 2019, Jowett and his team [68] proposed a new technology consisting of a neuroprosthetic—biocompatible—device (NPD) implanted on the zygomatic region of the paralyzed hemiface of the rat that evokes facial movements thanks to electromyography signals from epimysial electrode arrays (EEAs) implemented on the contralateral health hemiface. This technology should enable symmetric facial movements and expressions as well as avoid involuntary neural activities that lead to undesirable facial contractions thanks to high-frequency alternating current (HFAC).

Another promising device is electronic pacing, which has been much studied on animals in recent years [69]. In 2019, Makela et al. assessed the application of this device on humans [70]. The authors found that this device was able to simultaneously stimulate the frontalis, zygomaticus, orbicularis oculi, and orbiculari oris muscles even if the paralysis had occurred several years earlier. Obviously, the muscles should not be completely denervated. The main limit of this device is the pain related to stimulation, which was very variable from patient to patient.

Moreover, the study of nerve injury and the definition of the best therapeutic strategy are enhanced by advanced imaging techniques. The 3D high-resolution ultrasound (3D HRUS) and magnetic resonance microscopy (MRM) have shown significant potential in the study of the fascicular anatomy of peripheral nerves. In particular, the 3D HRUS provides non-invasive, real-time images with good spatial resolution, while MRM ensures a more accurate representation of the smallest and interfascicular connections [71]. Therefore, in PFP, these technologies could facilitate the assessment of nerve lesions and support intraoperative monitoring, helping to improve the accuracy of nerve repair techniques and optimize surgical outcomes.

## 7. Discussion

Peripheral facial palsy represents a social stigma. Indeed, PFP causes a disfigurement of the face with loss of harmony and symmetry as well as difficulty in performing daily facial movements and activities. Moreover, patients affected by PFP could develop synkinesis, too. Post-paralysis synkinesis is a disorder characterized by involuntary facial movements that accompany voluntary ones and is a frequent consequence of facial nerve damage. This disorder, associated with abnormal regeneration of the nerves, can cause uncoordinated movements of the face, such as unwanted muscle contractions during smiles or other expressions, with a negative impact on the patient’s quality of life, both from a functional and aesthetic point of view. Therefore, in PFP management, synkinesis should also be treated. There are several therapeutic strategies, such as physical rehabilitation, chemo-denervation by using botulinum toxin, selective neurectomy, selective myectomy, nerve transfers, and free muscle transfer [72]. Physical rehabilitation (e.g., Kabat technique, neuromuscular retraining) in combination with surgery ensures satisfactory results and faster healing and reduces the risk of synkinesis [73]. Shikara et al. demonstrated that selective neurectomy offers significant improvements in facial symmetry and muscle control. In addition, a reduction in the need for post-operative botulinum toxin treatments has been observed, suggesting that this technique may offer long-term results for patients with severe synkinesis [74]. On the other side, Ali et al. stated that most of these treatments require the repetition of the procedure and, in the case of neurectomy, the potential regrowth or accidental nerve injuries [75]. In their study on transgenic rat models, the authors proposed new therapies comparing the effectiveness of vincristine, a chemotherapeutic agent, with myelin-associated glycoprotein (MAG) in reducing the risk of aberrant facial nerve regeneration, as in synkinesis.

Since 1895, surgeons have attempted to find the best surgical technique that could reanimate the paralyzed facial side. Both static and dynamic strategies have been proposed and performed: the former is easier but with unsatisfactory results; the latter requires a long learning curve and more invasive surgeries but the best facial recovery [5]. Reviewing the literature, several treatment options have been performed over the years. McLaughlin [5] proposed the fascia lata graft because it is flat and resistant to tension; however, for the reanimation of the lower third of the face, dynamic techniques are preferred. Therefore, after McLaughlin, other myoplastic surgeries were performed by using the temporalis muscle flap [14,15,16,17,18], latissimus dorsi muscle flap [19,20,21,22,23], and sternohyoid muscle flap [24,25]. The first two techniques have been found to be effective in achieving both spontaneous and voluntary smiling after long rehabilitation (at least one year).

Regarding the upper third of the face, other techniques have been attempted: some using muscle flaps [27,28,29], others acting on the eyelid tarsal plate [30,31,32], and others on autologous grafts (e.g., conchal cartilage, dermis) [39,40]. All of these surgeries are static techniques and have proven effective in narrowing the palpebral fissure to treat the lagophthalmos and so, avoid complications such as epiphora, conjunctivitis, and corneal ulceration. Many authors suggest combining static and dynamic techniques to simultaneously treat both the upper and lower part of the paralyzed side of the face [76].

In some cases, facial nerve reconstruction with local and/or free nerve graft has been performed. Some of these, more commonly used, are the trifurcation approach [41,42,43,44,45,46,47,48,49,50], which uses the GAN and the hypoglossal nerve, the masseteric nerve transfer [51,52,53,54,55], and the cross-face nerve grafting [56,57,58,59,60]. The first represents the most widely used technique since it enables the best results with minimum comorbidity. On the contrary, masseteric nerve transfer does not enable spontaneous smile, and cross-face nerve grafting is related to the risk of lesions of facial nerves on the healthy facial side. As written above, in this surgery, a nerve interposition procedure could be performed: it could be a motor or sensory nerve. An animal study found no significant difference between the two types of nerve in terms of outcome, in contrast with previous in vitro studies [77,78,79].

The search for the best therapy is not over, and further studies have been conducted using the new bioengineering approaches. They include the use of a conduit embedded with growth factors (e.g., FGF-2, bone marrow stem cells, adipocytes) [62,63,64,65], the NPD [68], or the electronic pacing implanted in the paralyzed facial side [69]. Some nerve interface technologies have been developed [80]. The simplest includes only one electrode on the nerve, and the signal is inaccurate. The flat interface nerve electrodes (FINEs), with many electrodes, can selectively stimulate individual fascicles of the nerve with a better result. Recently, devices with implanted electrodes inside the nerve have also been developed; however, studies reported deterioration of the device’s performance within 6 months [81]. New technology includes the regenerative peripheral nerve interface (RPNI), which includes a muscle intermediary between the nerve and the electrode to avoid mechanical mismatch. Moreover, a preliminary study has been carried out regarding electroactive polymer artificial muscle [82]. In this context, to facilitate and improve neurophysiological studies on animals, an electronic implanted device has been developed; indeed, it is wireless, avoiding movement limitation or impairment of natural behaviors [83]. This technology could change the research on the nervous system, improving the reliability and accuracy of data collected. An animal study explored the effectiveness of a multi-channel bridge containing a lentivirus coding for interleukin-4 (IL-4), an anti-inflammatory protein. The authors found that this bridge promotes an environment conducive to nerve regeneration by reducing inflammation, a factor that often hinders full recovery after facial nerve injury [84]. Equally effective in promoting nerve regeneration in animal study has been the use of dental pulp stem cells, from which the authors have built scaffold-free ducts that promote axial regeneration and provide neurotrophic factors, mimicking the natural environment of the nerve [85].

## 8. Conclusions

The management of PFP is an important clinical challenge, requiring a multidisciplinary approach and personalized assessment for each patient. Facial resuscitation techniques, both static and dynamic, have undergone developments over the decades. Dynamic techniques, such as muscle transfer and nerve grafting, demonstrate superior results in terms of restoring voluntary and spontaneous facial movement, giving patients greater expression and function. However, the static techniques remain useful where dynamic options are not applicable, ensuring a partial aesthetic improvement. The choice of technique depends on many factors, including the patient’s age, the duration of the paralysis, the availability of suitable tissues or muscles for transplantation, and surgeon skill. Despite the progress made, no technique can be considered the gold standard for all patients. Each case of PFP must be treated with an individualized approach. May the future of facial resuscitation be the innovative solutions, such as tissue engineering and stem cell therapies?
